# Fungal Keratitis: Clinical Features, Risk Factors, Treatment, and Outcomes

**DOI:** 10.3390/jof8090962

**Published:** 2022-09-15

**Authors:** Sarah Atta, Chandrashan Perera, Regis P. Kowalski, Vishal Jhanji

**Affiliations:** 1 Department of Ophthalmology, University of Pittsburgh School of Medicine, Pittsburgh, PA 15213, USA; 2 Department of Ophthalmology, Stanford University School of Medicine, Stanford, CA 94305, USA; 3 The Charles T. Campbell Ophthalmic Microbiology Laboratory, University of Pittsburgh Medical Center, Pittsburgh, PA 15213, USA

**Keywords:** fungal keratitis, ophthalmic infection, antifungal, cornea

## Abstract

Fungal keratitis (FK) can be challenging to diagnose and treat. In this retrospective case series, FK cases presenting at the University of Pittsburgh Medical Center, Pennsylvania, USA, from 2015 to 2021 were reviewed for ocular risk factors, clinical presentation, management, and outcomes. Twenty-eight cases of FK were included. The median presenting age was 58.5 (18.5) years, and the median symptom duration prior to presentation was 10 (35.8) days. Predisposing ocular risk factors included contact lens use (67.9%), recent ocular trauma/abrasion (42.9%), and history of ocular surgery (42.9%). The median presenting visual acuity (VA) was 1.35 (1.72) LogMAR. About half presented with a central ulcer (42.9%), large infiltrate (6.7 (6.3) mm^2^), corneal thinning (50.0%), and hypopyon (32.1%). The majority of isolated fungal species were filamentous (75.0%). Most common antifungal medications included topical voriconazole (71.4%) and natamycin (53.6%) drops and oral voriconazole (64.3%). Surgical management was necessary in 32.1% of cases and enucleation in one case. Defect resolution occurred in 42.5 (47.0) days, and median final VA was 0.5 (1.84) LogMAR. Features associated with poor final visual outcomes included poor initial VA (*p* < 0.001) and larger defect size (*p* = 0.002). In conclusion, unlike prior studies in the northeast region of the USA, FK was commonly caused by filamentous fungi, and antifungal management most often consisted of topical and oral voriconazole.

## 1. Introduction

Fungal keratitis (FK) is an important cause of ocular morbidity due to resulting corneal scarring, and it accounts for about 40% to 50% of all microbial keratitis cases worldwide [1]. The incidence of FK is highest in developing countries [2], likely influenced by geographical location and socioeconomic status [1]. Over one-hundred fungal species have been shown to cause FK [3]. Within the United States, the most commonly isolated species include *Fusarium*, *Candida,* and *Aspergillus*, and studies have shown differences in the incidence of FK and species of isolates in warm, southern locations as compared to northern regions with cooler conditions [1]. FK was found to account for 6.7% of all infectious keratitis cases in a 14-year period at our eye center at the University of Pittsburgh [4].

Numerous risk factors have been associated with the development of FK, such as trauma, ocular surface disease, topical steroid use, and atopic disease [5,6,7]. Occupations involving agriculture and ocular exposure with vegetative matter are important risk factors for filamentary fungal infections, particularly in developing countries [1,5]. Contact lens use is a leading risk factor in developed countries [8], with a rise in FK cases paralleling the increased use of CL overtime [5]. Delays in the diagnosis of FK may occur due to challenges related to prolonged isolation time and negative cultures. Microscopic examination of corneal scrapings is typically used for preliminary diagnosis, followed by gold standard diagnostic testing with isolation on Sabouraud dextrose agar and blood agar, which is highly specific, but not sensitive [5].

Given the challenges in diagnosis, a high index of clinical suspicion is required to initiate timely management. In comparison to bacterial keratitis, fungal keratitis generally has worse clinical outcomes [9]. Even with appropriate diagnosis, management is challenging since many antifungal agents have poor penetration into the cornea [1]. Various antifungal management options have been described, including topical natamycin, topical amphotericin B, and topical and oral voriconazole [5,10,11]. The purpose of the present study is to characterize the clinical manifestations, management, and outcomes of fungal keratitis in this region in order to provide insights that will allow for timely diagnosis and initiation of optimal management. Given the geographic predominance of the disease, region-specific data are necessary to provide appropriate care.

## 2. Materials and Methods

This single-center, retrospective case series was approved by the Institutional Review Board of the University of Pittsburgh prior to data collection and is in compliance with the Health Insurance Portability and Accountability Act. Microbiological culture data from Charles T. Campbell Microbiology Laboratory were obtained for smear- or culture-proven cases of fungal keratitis. All cases of fungal keratitis at the University of Pittsburgh Medical Center (UPMC) Eye Center, a tertiary center, between 1 July 2015 and 30 November 2021 with accessible patient medical records, were included for analysis.

At our center, ocular samples from the cornea, conjunctiva, and lids are sent for microbiological testing for fungi isolation with routine culture media, including 5% sheep-blood-supplemented trypticase soy agar, chocolate agar, mannitol agar, and Sabouraud’s agar supplemented with gentamicin. The specimens are typically isolated within 3 to 7 days after inoculation. Microscopic examination of corneal scrapings by Giemsa or Gram staining were also obtained for each case. Viral testing for herpes simplex virus (HSV) and testing for Acanthamoeba were also performed in multiple cases in which there was clinical suspicion. For each case included in this study, microbiological data that were collected included the type of cultures performed, identification of the organism, and speciation of additional microbes if present.

Clinical data were collected from electronic medical records of each patient, including demographic information, ocular and systemic risk factors, symptom duration prior to presentation, presentation diagnosis, follow-up length, initial and final visual acuity, initial and final intraocular pressure (IOP), infiltrate size, infiltrate location, presence of hypopyon, presence of hyphema, medical management, adjunctive management, duration of treatment, and time to epithelial defect closure. Pittsburgh, Pennsylvania is located in the northeast USA and has a humid continental climate [12]. Given the relationship between fungal keratitis and environmental factors, the season at the time of presentation was collected as well. Clinical outcomes were defined by management strategies and final visual acuity. Visual acuity was extracted from chart review as Snellen fractions and converted to LogMAR in Excel using the formula outlined by Tiew et al. [13].

Python (v3.7.0) was used for statistical analysis, specifically the scientific packages pandas (v3.6), numpy (v1.18.5), and scipy (v1.4.1). To calculate the *p*-values for discrete and continuous variables, chi-squared tests and t-tests were used, respectively. A *p*-value of less than 0.005 was considered statistically significant. The python packages matplotlib (v3.2.2) and seaborn (v0.10.1) were used to generate the associated graphs and figures.

## 3. Results

A total of 31 cases of fungal keratitis were identified at our center between July 2015 and November 2021, and 28 cases were included for review. Two cases were lost to follow-up, and one case lacked comparable outcomes for analysis due to the inability to assess visual acuity given their non-verbal status. Findings were divided into initial clinical presentation, medical management, clinical outcomes, and microbiological information.

Thirteen patients in our analysis were female (46.4%, 13), and the median presenting age was 58.5 (18.5) years old with a range of 31 to 86 years. Clinical history and examination findings are included in Table 1. All 28 cases were unilateral, and the median duration of symptoms prior to presentation was 10 (35.8) days. Many patients had advanced clinical presentations with a median presenting visual acuity (VA) of 1.35 (1.72) LogMAR, central ulcer (42.9%, 12), large infiltrate (6.7 (6.3) mm^2^), corneal thinning (50.0%, 14), and hypopyon (32.1%, 9). All cases presented with at least one ocular predisposing factor, with contact lens (CL) use being the most common (67.9%, 19). Among those with CL use, 57.9% had poor CL hygiene (sleeping or showering in contact lenses, not cleaning or changing contact lenses as required). Other common ocular risk factors included recent ocular trauma/abrasion (42.9%, 12), history of corneal ulcer or keratitis (35.7%, 10), and history of ocular surface disease (32.1%, 9), such as dry eyes, anterior basement membrane dystrophy, herpes zoster ophthalmicus, ocular HSV, limbal stem cell deficiency, and band keratopathy. Other ocular risk factors included history of ocular surgery (42.9%, 12), topical steroid (32.1%, 9) or other topical medication (35.7%, 10) use such as anti-glaucoma drops or antibiotics, and water exposure to the eye (14.3%, 4). Past ocular surgeries included cataract surgery (17.9%, 5), penetrating keratoplasty (PKP) (7.1%, 2), laser in situ keratomileusis (LASIK) surgery (3.9%, 2), glaucoma laser surgery (7.1%, 2), and panretinal photocoagulation (3.6%, 1). The majority of patients were also found to have systemic comorbidities (78.6%, 22), with systemic atopy (57.1%, 16) and hypertension (35.7%, 10) being the most common.

The profiles of medical management and treatment outcomes are included in Table 2. All patients included in our cohort were managed on an outpatient basis. Each case was managed with a form of antifungal medication, with voriconazole drops being most commonly administered (71.4%, 20), followed by oral voriconazole (64.3%, 18) and topical natamycin drops (53.6%, 15). Amphotericin B drops were used in 28.6% of cases (8), and other oral antifungal medications included diflucan (17.9%, 5) and ketoconazole (3.6%, 1).

Interestingly, management of every case also included topical antibacterial drops over the course of treatment. Fluoroquinolone drops were most commonly administered (89.3%, 25), followed by fortified antibiotic drops (67.9%, 19). Antiviral medication was used in the management of over a third of the cases (35.7%, 10). Topical steroids were used in the initial management of 11 cases (39.3%) and later for anti-inflammatory control in 12 cases (42.9%). Adjunctive management was implemented in the majority of cases (78.6%, 22), most commonly including serum drops (32.1%, 9) and surgical management with PKP (32.1%, 9). Other common adjunctive treatments included bandage contact lens (28.6%, 8) and debridement (25.0%, 7). Many patients experienced complications during the management period (46.4%, 13), including corneal perforation, graft rejection, superinfection, and development of a fungal ball. One patient required enucleation. The median time to defect resolution was 42.5 (47.0) days, with a median treatment duration of 65.5 (46.3) days. The median final VA was 0.5 (1.84) LogMAR.

Fungal keratitis was proven by culture positivity in 75% of cases (21) (Table 3), while the remainder were managed based on clinical suspicion. Microbial smear was collected in all 28 cases and showed evidence of fungal elements in half (50%, 14). Fungal cultures were collected in the majority of cases (92.9%, 26) and resulted in positive cultures in slightly over half (57.7%, 15). Corneal and conjunctival cultures were also collected in several cases and were positive for fungus in 32% (8/25) and 10.5% (2/19) of cases, respectively. There was concurrent bacterial infection in half of the patients (50.0%, 14) and concurrent HSV infection in one patient. Coagulase negative staphylococcus was identified in 12 cases (42.9%). Microbial cultures revealed a wide range of isolated species of fungus. Among the 20 cases that included identifiable fungal features on culture, 75.0% were filamentous (15/20), while 25% were yeast (5/20). Specific isolates included *Aspergillus* (10.7%, 3), *Fusarium* (10.7%, 3), *Candida* (7.1%, 2), *Acremonium* (7.1%, 2), *Scedosporium* (7.1%, 2), *Bipolaris* (3.6%, 1), *Alternaria* (3.6%, 1), and *Exserohilum* (3.6%, 1). Six cases only included unspecified descriptions, such as “yeast” (10.7%, 3), “hyphae” (7.1%, 2), and “fungal elements” (3.6%, 1).

**Figure 1:**
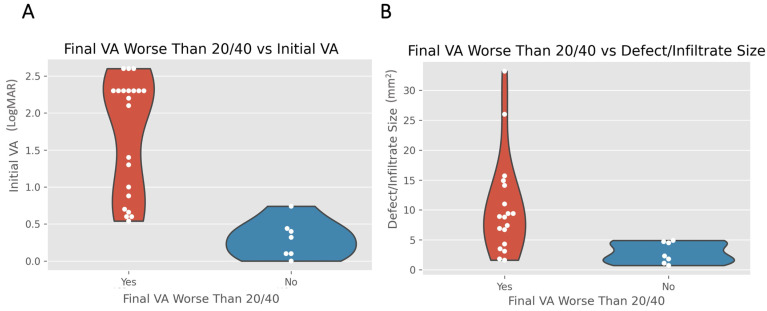
Worse initial visual acuity (**A**) and larger defect size (**B**) were significantly associated with worse final visual outcomes (*p* < 0.005). VA: visual acuity; LogMAR: logarithm of the minimum angle of resolution.

In order to assess the association of the various features with the clinical outcomes of the patients with fungal keratitis, the cohort was divided into “better” or “worse” visual acuity with a cutoff of 20/40 (0.30 LogMAR) (Table 4). Two features that were found to have a statistically significant association with worse final visual outcomes were presenting with a worse initial visual acuity (*p* < 0.001) and presenting with a larger defect size (*p* = 0.002) (Figure 1). Several features were found to be less strongly associated with a worse final visual acuity, including presentation during cooler months of fall and winter (*p* = 0.044), presentation with a central defect (*p* = 0.027), having a negative smear result (*p* = 0.027), and receiving oral voriconazole during medical management (*p* = 0.038). Features that were associated with a better visual outcome at final evaluation included presentation during the warmer months of spring and summer (*p* = 0.044), having a positive smear result (*p* = 0.009), presence of *Aspergillus* on culture (*p* = 0.039), and receiving adjunctive treatment with debridement (*p* = 0.030).

## 4. Discussion

This study included 28 cases of fungal keratitis, 75% culture-positive, that were treated at the UPMC Eye Center between 2015 and 2021. Fungal keratitis is less prevalent in the northern states [14], but can have significant effects on visual function. Our study found that the median symptom duration prior to presentation was long (10 days), infiltrate sizes at presentation were large (6.7 mm^2^), and corneal thinning was present in half. Complications during the management period occurred in 46% and included corneal perforation, graft rejection, superinfection, and development of a fungal ball. The median visual acuity from presentation to final visit improved from 1.35 (about 20/450) to 0.5 (about 20/60) LogMAR.

The most common ocular risk factor for FK identified in our study was the use of contact lenses, which is a known risk factor for fungal keratitis. It has been documented that CL-related FK may confer a more favorable prognosis as compared to other causes [15]. Furthermore, a majority of contact lens users endorsed poor contact lens hygiene (57.9% of CL users in this series), which further increases the risk of fungal keratitis [16]. The mechanism of CL-associated fungal keratitis is thought to be related to the increased adherence of microbes to the cornea via the contact lens and also microenvironment changes in oxygen levels [17]. Over half of the patients with a history of CL use also reported an abrasion or trauma resulting in a foreign body within the eye.

A history of ocular trauma and exposure to vegetative materials among agricultural workers has been commonly cited as a risk factor for fungal keratitis in other studies [5,14]. Nine of the 12 patients who had a history of recent trauma or abrasion prior to presentation of FK reported the introduction of a foreign body to the eye after contact with vegetative material from outdoors, such as a garden or construction site. Others reported non-specific scratches to the eye or chemical injuries.

Topical steroid use has also been described as an ocular risk factor commonly associated with the worsening of fungal keratitis [5,9]. Corticosteroids are thought to impact the pathogenesis of FK by allowing fungi to easily replicate and decrease the effects of antifungal drugs that have limited corneal penetration [18]. However, a history of topical steroid use in the present study was interestingly not significantly associated with worse outcomes.

Overall, fungal keratitis is less common in developed countries [5], and at our institution, we have determined that FK makes up about 6.7% of infectious keratitis cases [4]. The geographical distribution and climate have been described to affect the presentation of FK, with potential increases in cases when warm climates better support fungal growth [14]. Our study revealed more cases of fungal keratitis from warmer months of the year (60.7%), which may support this theory. Of note, our analysis identified that cases of FK that presented during warm months generally had better visual outcomes as compared to FK presenting during cooler months. This could be related to the prevalence of different fungal species in particular climates that may have varying effects, such as a higher proportion of yeasts in the colder environments [14]. Additionally, our study consisted predominantly of cases caused by filamentous fungi, which are more frequent in warmer climates [14].

*Aspergillus* and *Fusarium* were equally greatest in frequency (three each) among the specific microbial species identified in the present study. Interestingly, these filamentous species are typically more common in tropical and subtropical regions, such as Miami [5,14]. However, one study in Minnesota identified that the majority of FK cases were growing filamentous species among patients who were farmers or exposed to outdoor ocular trauma [19], similar to the findings in our study. Although *Candida* was the most common isolated fungal species observed in northern cities, including Philadelphia [7] and New York City [6,20], only two cases with isolated *Candida* were identified in the present case series. However, three other cases included unspecified yeast elements, which may represent *Candida*.

In comparison to studies of bacterial keratitis at our center, we found that symptom duration prior to presentation was relatively longer in FK, which had a median symptom duration of 10 days compared to 5 days in methicillin-resistant *Staphylococcus aureus* (MRSA) keratitis [21] and 4 days in *Serratia* keratitis [22]. This difference could be related to the difficulty in establishing the diagnosis of fungal keratitis and the difficulty in providing a timely referral given that FK is less common than other forms of microbial keratitis. One study found that clinicians were only able to accurately identify fungal keratitis cases in 38% (5 of 13) of the FK cases presented to them [23]. This underscores the importance of familiarity with clinical features to establish a timely diagnosis.

Antifungal management with natamycin has previously been described as an effective drug to treat FK [10], with amphotericin B 0.3–0.5% and voriconazole 1% being effective alternatives [5]. A benefit of topical natamycin over topical voriconazole in the management of FK, particularly that caused by *Fusarium*, was documented in the randomized controlled Mycotic Ulcer Treatment Trial (MUTT) I [24]. Natamycin has also been shown to be associated with better visual outcomes and less likelihood of corneal perforation with the need for PKP as compared to voriconazole in fungal keratitis [25]. However, natamycin has been described to have poor entry to the corneal stroma with reports of treatment failure [5,10]. While voriconazole has been shown to have a broad spectrum of activity against various fungal species, including *Candida*, *Aspergillus*, *Scedosporium*, and *Fusarium* [18], the MUTT II study concluded that there is no apparent benefit in adding oral voriconazole to topical antifungal agents in the management of filamentous FK [26]. However, our study consisted mainly of FK caused by filamentous fungi and was most commonly managed with topical and oral voriconazole (71.4% and 64.3%, respectively). A previous case of fungal keratitis by *Fusarium* at our center was reported to have significant improvement after transition from topical natamycin 5%, amphotericin B 0.15%, and intracameral amphotericin B to topical and oral voriconazole [11]. Further study may be necessary given the conflicting evidence between various antifungal management options.

With regards to adjuvant therapy, surgical intervention with PKP is five- to six-times more likely in FK as compared to bacterial keratitis [14]. The percent of patients in the present series that required surgical intervention with PKP (32.1%) was similar to that of other studies, such as a retrospective case series of fungal keratitis infections treated at Duke University Eye Center (37%) [27] and at Wills Eye Hospital (25%) [7]. Although the study by Afshari et al. [27] found that the rate of surgical intervention was highest in patients with previous PKP, this was not the case in our series in which only one patient with a prior PKP required repeat PKP.

Although similar to the sizes of previous retrospective studies on fungal keratitis, a limitation of the present study is the sample size. Given the relatively lower incidence of FK in our patient population as compared to other regions, the power of the study is limited. Retrospective studies have selection bias and misclassification errors. Our hospital is a tertiary care referral center with the likelihood of a disproportionate number of non-routine keratitis cases. There may also be a component of referral bias with our center seeing more severe forms of keratitis after initial management elsewhere, resulting in worse outcomes after delays.

## 5. Conclusions

In conclusion, our study suggests that although fungal keratitis is less common than other forms of microbial keratitis in this region, it is an important cause of visual impairment and necessitates prompt diagnosis based on clinical suspicion. Unlike previous studies in the northeast region, FK was most commonly caused by filamentous fungi and responded to management with topical and oral voriconazole. Challenges with the isolation of fungal elements in microbiological culture can result in delays in diagnosis and appropriate management. Future study of the management of FK with voriconazole as opposed to the common use of natamycin may warrant further study.

## Figures and Tables

**Table 1:** Clinical profile of patients with fungal keratitis at presentation.

Feature	N = 28
*Demographic/General Information*	
Female (%, n)	46.4 (13)
Age at presentation (median, IQR)	58.5 (18.5) years
Laterality (left eye) (%, n)	57.1 (16)
Symptom duration prior to presentation (median, IQR)	10 (35.8)
Referred from outside provider (%, n)	78.5 (22)
Warm season (spring/summer months) (%, n)	60.7 (17)
*Ocular Risk Factors* (%, n)	
Contact lens use	67.8 (19)
Poor contact lens hygiene	57.8 (11/19)
Recent ocular trauma/abrasion	42.8 (12)
Water exposure to eye	14.2 (4)
History of:	
Corneal ulcer/keratitis	35.7 (10)
Ocular surface disease *	32.1 (9)
Glaucoma	10.7 (3)
Ocular surgery	42.8 (12)
PKP	7.1 (2)
Cataract surgery	17.8 (5)
LASIK surgery	17.8 (5)
Glaucoma/retina surgery	10.7 (3)
Topical steroid use	32.1 (9)
Topical medication use	35.7 (10)
*Systemic Comorbidities* (%, n)	
Hypertension	35.7 (10)
Smoker	21.4 (6)
Systemic Atopy	57.1 (16)
*Clinical Features at Presentation*	
Initial visual acuity (median, IQR)	1.35 (1.72)
Initial intraocular pressure (median, IQR)	13.0 (8.0) mmHg
Central ulcer (%, n)	42.8 (12/28)
Infiltrate size (median, IQR)	6.7 (6.3)
Corneal thinning (%, n)	50.0 (14/28)
Hypopyon (%, n)	32.1 (9/28)

N: number of patients; IQR: interquartile range; PKP: penetrating keratoplasty; LASIK: laser-assisted in situ keratomileusis. * History of ocular surface diseases: dry eyes, ABMD, HZO, ocular HSV, LSCD, band keratopathy.

**Table 2:** Management and outcomes of patients with fungal keratitis.

Feature	N = 28
*Medical Management* (%, n)	
*Anti-fungal*	
Natamycin drops	53.5 (15)
Amphotericin B drops	28.5 (8)
Intrastromal amphotericin B	10.7 (3)
Voriconazole drops	71.4 (20)
Oral voriconazole	64.2 (18)
Intrastromal voriconazole	17.8 (5)
Intravitreal voriconazole	3.5 (1)
Oral ketoconazole	3.5 (1)
Oral diflucan	17.8 (5)
*Anti-bacterial*	
Fluoroquinolone drops	89.2 (25)
Gatifloxacin	17.8 (5)
Moxifloxacin	67.8 (19)
Ciprofloxacin	21.4 (6)
Ofloxacin	25.0 (7)
Besifloxacin	17.8 (5)
Fortified drops	67.8 (19)
Fortified tobramycin	67.8 (19)
Fortified cefazolin	57.1 (16)
Fortified vancomycin	21.4 (6)
*Anti-viral*	
Valacyclovir	32.1 (9)
Acyclovir	3.5 (1)
Ganciclovir	7.1 (2)
*Other*	
Any steroids	64.2 (18)
Topical steroids prior to anti-fungal	39.2 (11)
Topical steroids later in management	42.8 (12)
Vitamin C	64.2 (18)
Doxycycline	71.4 (20)
Cyclopentolate	71.4 (20)
Atropine	25.0 (7)
*Adjunctive Management* (%, n)	
Any adjunctive treatment	78.5 (22)
Serum drops	32.1 (9)
Bandage contact lens	28.5 (8)
Tarsorrhaphy	7.14 (2)
Debridement	25.0 (7)
PKP	32.1 (9)
Enucleation	3.5 (1)
Other *	14.2 (4)
*Clinical Outcomes*	
Treatment duration (median, IQR)	65.6 (46.3) days
Time to defect resolution (median, IQR)	42.5 (47.0) days
Final visual acuity (median, IQR)	0.5 (1.84) LogMAR
Final intraocular pressure (median, IQR)	12.5 (6.5) mmHg
Complication during management (%, n)	46.4 (13)

N: number of patients; IQR: interquartile range; LogMAR: logarithm of the minimum angle of resolution; mmHg: millimeters of mercury. * Other adjunctive management: glue, amniotic membrane, needle diathermy, synechiolysis.

**Table 3:** Microbiological profile of patients with fungal keratitis.

Microbiology Collection Type, Findings	Percentage (n), N = 28
Gram stain collected	10.7 (3)
with fungal elements	33.3 (1/3)
Smear collected (corneal)	100 (28)
with fungal elements	50.0 (14/28)
Fungal culture collected	92.8 (26)
Positive culture for fungus	57.6 (15/26)
Corneal culture collected	89.2 (25)
Positive culture for fungus	32.0 (8/25)
Conjunctival culture collected	67.8 (19)
Positive culture for fungus	10.5 (2/19)
Total culture-positive cases	75 (21)
Concurrent bacterial infection	50.0 (14)
Concurrent herpes simplex virus infection	3.5 (1)
*Isolated fungus types*	
Filamentous	
*Aspergillus* spp.	10.7 (3)
*Fusarium* spp.	10.7 (3)
*Acremonium* spp.	7.1 (2)
*Scedosporium* spp.	7.1 (2)
Unspecified “hyphae”	7.1 (2)
*Alternaria* spp.	3.5 (1)
*Bipolaris* spp.	3.5 (1)
*Exserohilum* spp.	3.5 (1)
Yeast	
Unspecified “yeast”	10.7 (3)
*Candida* spp.	7.1 (2)
Unspecified “fungal elements”	3.5 (1)

**Table 4:** Features on clinical presentation that were significantly different between patients with a poor visual outcome (VA less than 20/40) vs. patients with a better visual outcome (VA greater than 20/40).

Clinical Feature	Poor VA Outcome	Better VA Outcome	*p*-Value
Presentation			
Cool season at time of presentation (fall/winter) (%, n)	52.3% (11/21)	0% (0/7)	0.044
Warm season at time of presentation (spring/summer) (%, n)	47.6% (10/21)	100% (7/7)	0.044
Initial VA (mean ± SD)	1.7 ± 0.8 LogMAR (21/28)	0.3 ± 0.3 LogMAR (7/28)	<0.001
Defect size (mean ± SD)	10.3 ± 8.0 (18/28)	2.8 ± 1.6 (7/28)	0.002
Central defect location (%, n)	57.1% (12/21)	0% (0/7)	0.027
Microbiology			
Smear-positive for fungal elements (%, n)	33.3% (7/21)	100% (7/7)	0.009
Smear-negative for fungal elements (%, n)	57.1% (12/21)	0% (0/7)	0.027
*Aspergillus* on culture (%, n)	0% (0/21)	42.8% (3/7)	0.039
Treatment			
Oral voriconazole (%, n)	80.9% (17/21)	14.2% (1/7)	0.038
Debridement (%, n)	9.5% (2/21)	71.4% (5/7)	0.030

SD: standard deviation; VA: visual acuity; LogMAR: logarithm of the minimum angle of resolution.

## Data Availability

Not applicable.
